# Use of Topical Oxygen Therapy to Optimize Postoperative Healing of Oral Surgical Wounds After Dental Implant Placement

**DOI:** 10.1155/crid/6159662

**Published:** 2026-05-15

**Authors:** Reyna María Ocegueda Estrada, Fabio Luiz Andretti, Tatiana Miranda Deliberador

**Affiliations:** ^1^ Implantology Department, Latin American Institute of Dental Research and Education–ILAPEO, Curitiba, Paraná, Brazil; ^2^ Department of General Dentistry, University of Tennessee Health Sciences Center College of Dentistry, Memphis, Tennessee, USA

**Keywords:** dental implants, oral wound healing, topical oxygen therapy

## Abstract

Implant‐supported and implant‐borne prostheses have become a common alternative for treating total or partial edentulous patients with good long‐term results. Prompt and effective healing of surgical wounds will contribute to this purpose. The objective of this case report was to evaluate by clinical observation the healing of oral postsurgical wounds following dental implant placement with or without the use of oral gel with active oxygen (BlueM). Two patients underwent dental implant placement surgery by conventional open techniques to evaluate the healing of postsurgical wounds subjected to flaps, both with immediate loading and provisionalization. In the first one, a full‐arch surgery with four implants and bone reduction was performed. In the second, two mandibular single implants were placed in Sites 46 and 36 with immediate loading and provisionalization. The active oxygen gel product (BlueM) was placed in the tissues, only on the right side, before closing the flap and after suturing. Patients were instructed to place the product only on the right side three times a day, and no product or topical medication on the left side. Observational comparisons were made by the early wound healing index (EHI) at 7, 15, and 30 days after surgery in the complete arch and at 3, 7, 15, and 30 days in the single cases. Within the limitations of these clinical cases and based solely on clinical observations, the use of active oxygen oral gel (BlueM) may enhance the healing of surgical wounds following dental implant placement, and literature supports its antiseptic and bactericidal properties. The oral gel with active oxygen (BlueM) may represent a reliable adjunctive approach for the postoperative care of oral wounds following dental implant placement. In these clinical cases, enhanced wound healing was observed; however, further controlled clinical studies are required to substantiate these findings.

## 1. Introduction

Treatment with osseointegrated dental implants is a predictable and widespread treatment alternative that can eliminate the need for fixed dentures or removable prostheses in the rehabilitation of edentulous patients, with predictable short‐ and long‐term success rates [1]. The factors that determine the success and survival of implants are related to the host, the implant, the prosthesis, and the surgery [2]. Regarding the surgical aspect, the healing of the surgical wound is critical for reestablishing the protective barrier against pathogenic invaders, mechanical stresses, and physical trauma [3]. Four consecutive stages of tissue change occur in this healing process: hemostasis, inflammation, proliferation, and remodeling. Hemostasis and inflammation begin at the time of injury and continue for up to 6 days. The proliferation stage involves reepithelialization, angiogenesis, granulation tissue formation, and collagen deposition. This phase starts around Day 4 and can last for up to 3 weeks after a soft tissue injury. The soft and/or hard tissue remodeling phase will continue for approximately 1 year [4]. Wound healing in the mouth is driven by the inflammatory and vascular response, in which angiogenesis or neovascularization plays a significant role. Capillary growth is necessary for optimal wound healing by providing oxygen and micronutrients and removing catabolic waste products from the healing tissues [5].

Therapeutic approaches aimed at improving tissue oxygenation may be the key to success in wound management [6]. Oxygen is an essential element for the maintenance of healthy tissues and for wound healing processes [7]. While wound hypoxia is a natural consequence of tissue injury, it requires an adequate supply of oxygen to provide the energy needed for neovascularization and epithelialization [3, 7]. Both hyperbaric oxygen therapy and topical oxygen therapy have been reported to improve wound healing [6]. Other methods promote angiogenesis in oral wound healing, such as ultrasound, lasers, platelet‐rich plasma (PRP)/platelet‐rich fibrin (PRF), and various chemical agents such as hyaluronic acid, anthoxanthin, and *Centella asiatica*. These include cutting‐edge therapies like the use of growth factors, bioengineered skin substitutes, and stem cell therapy [3].

A product is currently on the market (BlueM) that is focused on the principle of improving topical oxygen delivery to oral wounds. This easy‐to‐use product seems to positively influence tissue healing by improving oxygenation without risking the stability of the microbiome. The slow and continuous release of oxygen from BlueM is facilitated by its formula, which contains honey (with the glucose oxidase enzyme) and sodium perborate. When these ingredients meet tissue fluids, they are transformed into low concentrations of hydrogen peroxide (H_2_O_2_) (0.003%–0.15%). The oral gel is the product with the highest level of oxygen release, which enhances its healing and bactericidal actions [3, 8].

Recently, clinical [9] and animal research [8], as well as review articles [10], have shown the benefits of using this oral gel in treating periodontitis in diabetic patients [9] and in promoting tissue healing [8, 10]. The use of topical oxygen therapy leads to better healing due to its bactericidal effect and its action on the formation of new blood vessels (stimulates angiogenesis) and collagen fibers [8].

The objective of this case report was to evaluate by clinical observation the healing of oral postsurgical wounds following dental implant placement with or without the use of oral gel with active oxygen (BlueM).

## 2. Case Presentation

### 2.1. Patient #1

#### 2.1.1. Diagnosis and Treatment Plan

**Figure 1:**
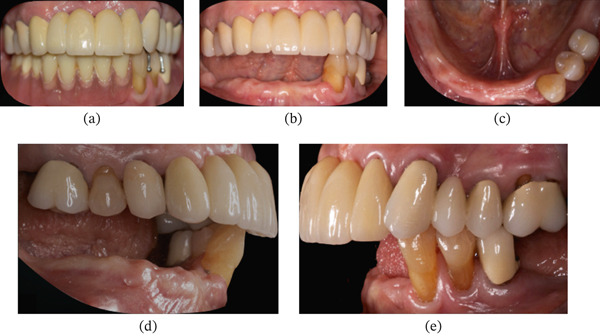
Initial clinical condition of Patient #1 (E.S., 72 years old, female). (a) Frontal view of the mandibular RPD. (b) Frontal view without the mandibular RPD. (c) Occlusal view of the lower arch. (d) Right lateral view. (e) Left lateral view.

A 74‐year‐old, systemically healthy female patient presented to the clinic seeking a fixed treatment option to replace her lower removable prosthesis and improve functionality. The upper arch had a fixed prosthesis on her natural teeth, and the lower arch was a Kennedy Class I with only three remaining teeth on the left side (Figure 1).

**Figure 2:**
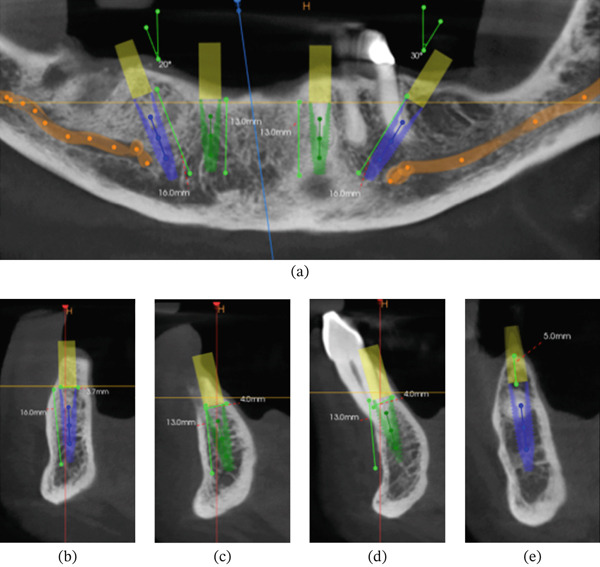
Panoramic and cross‐sectional views of the planned implants for Patient #1. (a–e) Two 13 × 3.75‐mm and two 16 × 3.75‐mm implants were installed in the lower arch.

After a comprehensive clinical and tomographic examination (Figure 2), the treatment plan consisted of a full‐arch protocol on four mandibular implants with immediate provisionalization and loading (NeoArch technique, Neodent), involving vertical bone reduction and a conventional open surgical technique to evaluate healing in extensive wounds. The oral gel with active oxygen (BlueM) was applied to the implants only on the right surgical site before and after flap closure. The patient was instructed to apply the gel three times a day only on the right side during the healing period to compare with the left side (no gel application—control side), where no topical product or medication was indicated. Clinical observations were made at 7, 15, and 30 days after surgery.

#### 2.1.2. Surgical Procedure

**Figure 3:**
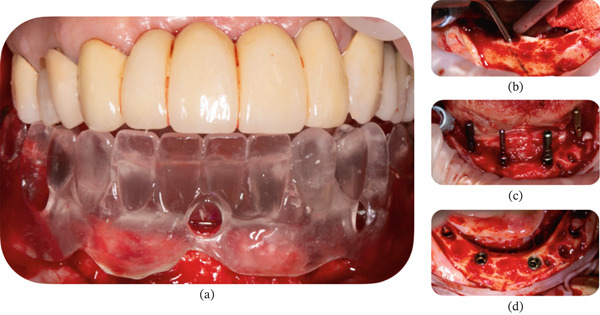
(a) Seating of the multifunctional guide after extraction of the failing remaining mandibular teeth and raising of the full‐thickness flap. (b) Location of the mental foramen with pencil marking to evaluate the relationship between the distal implants and the required angulation. (c) Checking parallelism between implants. (d) Four implants were delivered in the mandibular arch.

The surgical procedure began with asepsis and antisepsis, followed by the administration of a local anesthetic via right and left mandibular regional blocks with mepivacaine–epinephrine 36/0.018 mg, 1.8 mL (Scandonest 2% Special, Septodont, France). Extractions were performed on Teeth #33, #34, and #35. An incision was made along the entire alveolar ridge, from the right first molar to the left first molar region, with two posterior vestibular releasing incisions using a 15C scalpel blade. A full‐thickness flap was raised, confirming the seating of the multifunctional guide (Figure 3a). The planned osteotomy was performed with surgical gouges and drills, and the emergence of the right and left mental nerves was visually located and marked with sterile graphite (Figure 3b). The complete drilling sequence for the posterior implants was performed, with emergences designed at the level of the second premolars. The posterior implants were placed with an inclination of approximately 17° (Figure 3c,d). The implants (Helix GM Acqua, Neodent, Curitiba, PR, Brazil) were installed according to the manufacturer′s recommendations. All implants achieved a torque of more than 32 N·cm.

The transmucosal prosthetic attachments were then placed as follows: a conical angulated mini‐abutment GM exact 17° (Neodent) on the right and left posterior implants with a torque of 20 N·cm and a straight conical mini‐abutment GM (Neodent) on the right and left anterior implants with a torque of 32 N·cm.

**Figure 4:**
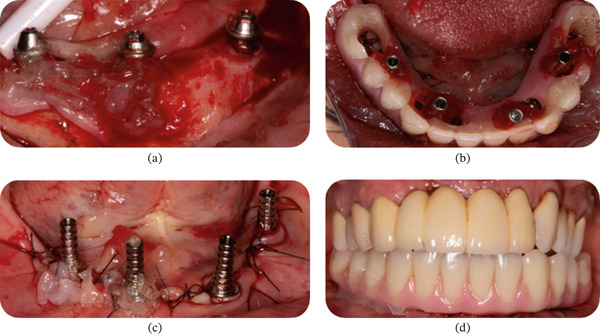
(a) Active oxygen gel (BlueM) was placed on the surgical site of the implants on the right side after suturing. (b) Pick‐up of the provisional full‐arch fixed restoration. (c) Application of active oxygen gel (BlueM) only on the right side after suturing, delivery of the impression copings, and pick‐up of the provisional full‐arch restoration. (d) Provisional full‐arch restoration supported on four implants. The patient was recommended to apply the oral gel at home every 8 h only on the right side.

Active oxygen gel (BlueM) was applied to the surgical site only on the right side (Figure 4a), and the wound was closed with eight single stitches using 4‐0 nylon suture. The maneuvers for the capture and adjustment of the provisional denture were performed immediately on four temporary titanium cylinders for the GM mini conical abutment (Figure 4b). Before placing the adjusted and polished provisional prosthesis, active oxygen gel (BlueM) was applied again to the sutured wound on the right side (Figure 4c).

The patient was prescribed amoxicillin/clavulanic acid 875/125 mg every 12 h for 7 days and ibuprofen 400 mg every 8 h for 3 days. As part of the postsurgical instructions, the patient was instructed to apply the active oxygen gel (BlueM) three times a day to the vestibular and lingual aspects only of the right‐sided wound every day until the end of the healing follow‐up period (Figure 4d).

### 2.2. Patient #2

#### 2.2.1. Diagnosis and Treatment Plan

**Figure 5:**
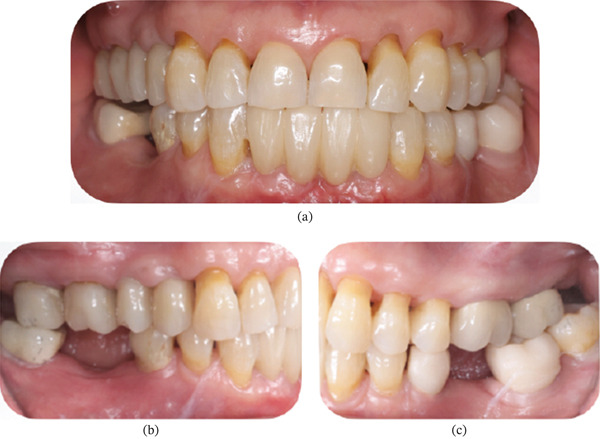
Initial clinical condition of Patient #2 (G.R.C., 67 years old, female). (a) Frontal view. (b) Right lateral view. (c) Left lateral view.

**Figure 6:**
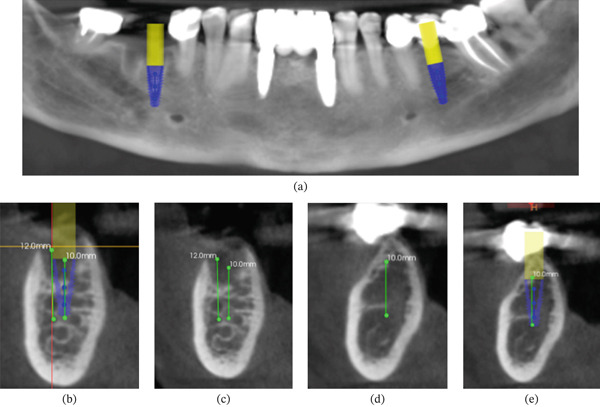
Panoramic and cross‐sectional views of the planned implants for Patient #2. (a–e) Two implants (3.75 × 10 mm on the left side and 4.3 × 10 mm on the right side) (Helix GM Acqua, Neodent) were delivered in the mandibular arch.

A 67‐year‐old, systemically healthy female patient presented for a consultation to receive fixed prosthetic teeth in edentulous Sites #46 and #36 after her diagnostic examination and evaluation (Figure 5). After a comprehensive clinical and tomographic examination, the treatment plan was to place two osseointegrated dental implants in these sites with immediate provisionalization and loading (Figure 6). The implants were placed with a conventional freehand surgical technique to evaluate healing after creating wounds with flaps.

The active oxygen extended‐release gel (BlueM) was applied only to the implant and the right surgical site before and after flap closure. The patient was instructed to apply the gel topically three times a day, only on the right side during the healing period, to compare with the left side, where no topical product was indicated. Clinical observations were made at 3, 7, 15, and 30 days after surgery. The left side was not treated with oral gel and served as a control side for observational comparison of clinical healing.

#### 2.2.2. Surgical Procedure

The surgical procedure began with asepsis and antisepsis, followed by local anesthesia administered via right and left mandibular regional blocks with mepivacaine–epinephrine 36/0.018 mg, 1.8 mL (Scandonest 2% Special France Septodont). Surgeries on the right and left sides were performed on the same day by the same operator.

**Figure 7:**
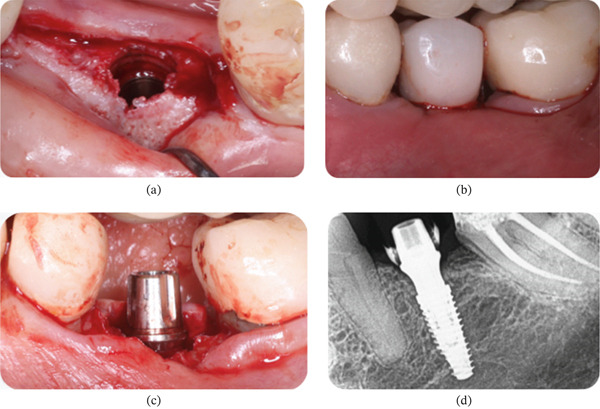
Patient #2 (left side). (a, b) Lateral view of the 2‐mm intraosseous implant (Universal Click GM Exact, Neodent) delivered. (c) After delivery of the provisional restoration. (d) Periapical radiograph taken immediately after the delivery of the provisional restoration.

On the left side, an incision was made along the alveolar ridge corresponding to Site #36, extending sulcularly to the proximal faces of the neighboring teeth. A full‐thickness flap was raised, and a drilling sequence was performed up to a 3.5‐mm drill. The area had low‐density medullary bone, so a Helix GM Acqua 3.75 × 10‐mm implant (Neodent, Curitiba, PR, Brazil) was placed 2 mm intraosseously from the vestibular crest (Figure 7a) with a torque of 32 N·cm. A universal Click GM Exact 4.5 × 4 × 3.5 abutment (Neodent, Curitiba, PR, Brazil) was installed with a torque of 20 N·cm (Figure 7b). The wound was sutured at the proximal papillae with polyglactin 910 4‐0, and a temporary prosthesis was placed in subocclusion without cement using a temporary click cylinder (Figure 7c). An immediate periapical X‐ray was taken to verify the correct position of the implant and the adaptation of the prosthetic component (Figure 7d).

**Figure 8:**
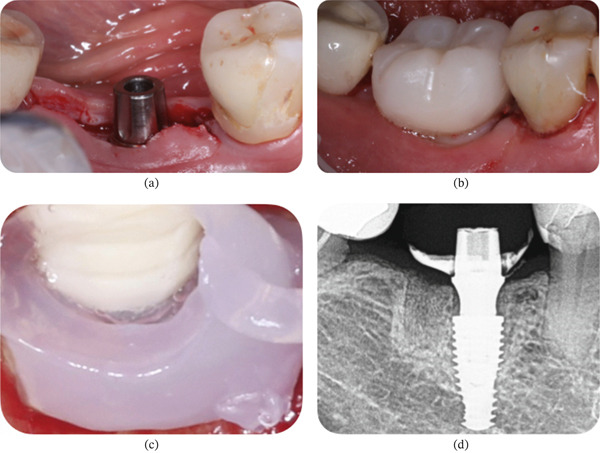
Patient #2 (right side). (a) Lateral view after the delivery of the abutment (Universal Click GM Exact). (b) Close‐up view of the surgical site after application of oral gel with active oxygen (BlueM) before closing the flap. (c) Lateral view after delivery of the provisional restoration. (d) Periapical radiograph taken immediately after the procedure.

On the right side, a similar incision was made along the alveolar ridge corresponding to Site #46. A full‐thickness flap was raised, and after a complete drilling sequence, a Helix GM Acqua 4.3 × 10‐mm implant (Neodent, Curitiba, PR, Brazil) was placed 2.5 mm intraosseously to the vestibular ridge with a torque of 32 N·cm. A Universal Click GM Exact 4.5 × 4 × 3.5 abutment was installed with 20 N·cm torque (Neodent, Curitiba, PR, Brazil) (Figure 8a). Afterward, active oxygen gel (BlueM) was applied to the surgical area (Figure 8b). The wound was sutured with stitches at the proximal papillae with polyglactin 910 4‐0, and a temporary prosthesis was placed in subocclusion (Figure 8c). An immediate periapical X‐ray was performed to verify the correct implant position and component adaptation (Figure 8d). At the end of the surgery, the oral gel with active oxygen was applied again.

The patient was prescribed oral amoxicillin 500 mg every 8 h for 7 days and ibuprofen 400 mg every 8 h for 3 days. As postsurgical instructions, the patient was asked to apply the active oxygen gel (BlueM) three times a day to the vestibular and lingual aspects of the wound only on the right side every day until the end of the healing follow‐up.

### 2.3. Healing Assessment

#### 2.3.1. Early Wound Healing Index (EHI)

Postoperative healing was assessed using the EHI [11], which differentiates between five different degrees as described below. For this evaluation, four blind and calibrated examiners were used. Each examiner received a document with postoperative photos and was instructed to carry out the EHI assessment three times in different weeks to ensure reliability. The final scores were determined by the values most frequently cited by four examiners across the three evaluation periods:

• EHI 1: complete flap closure, no fibrin line in the interproximal area.
• EHI 2: complete flap closure, fine fibrin line in the interproximal area.
• EHI 3: complete flap closure, fibrin clot in the interproximal area.
• EHI 4: incomplete flap closure, partial necrosis of the interproximal tissue.
• EHI 5: incomplete flap closure, complete necrosis of the interproximal tissue.

##### 2.3.1.1. Healing of Patient #1

**Figure 9:**
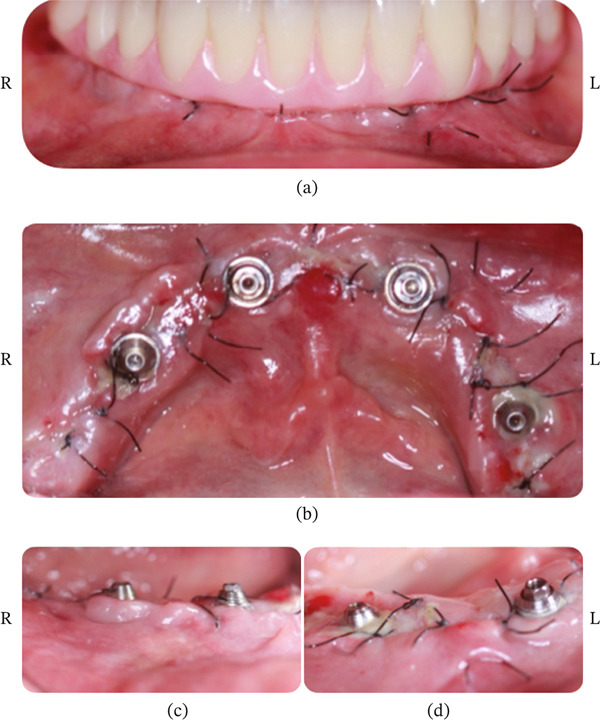
Patient #1 (7 days after operation) (L = left side; R = right side). (a) Image with the provisional full‐arch restoration in place. (b) Occlusal view after removal of the provisional restoration. It is observed that the right side that received the BlueM oral gel presents better healing. (c) On the right side, complete closure of the flap with a fine fibrin line in the interproximal area was observed. (d) On the left side, closure of the flap with a fibrin clot in the interproximal area was seen.

**Figure 10:**
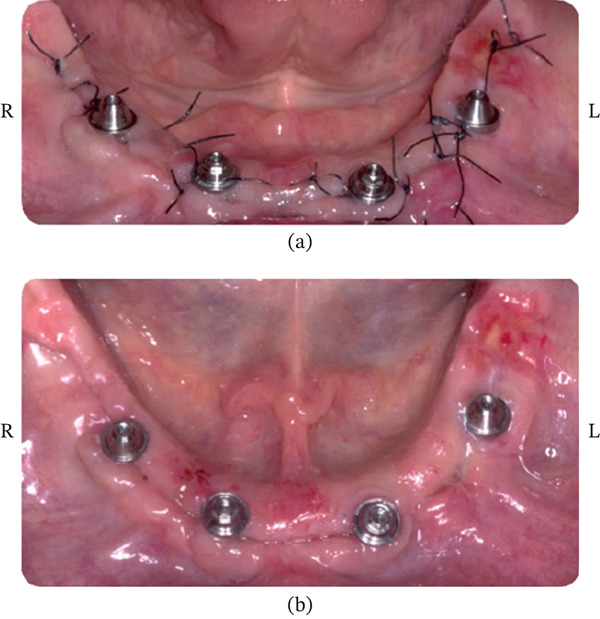
Patient #1 (15 days after operation) (L = left side; R = right side). (a) Occlusal view before suture removal shows the right side with better healing than the left side. (b) Occlusal view after suture removal shows the right side with full flap closure without fibrin zones. The left side shows full flap closure with thin fibrin zones.

**Figure 11:**
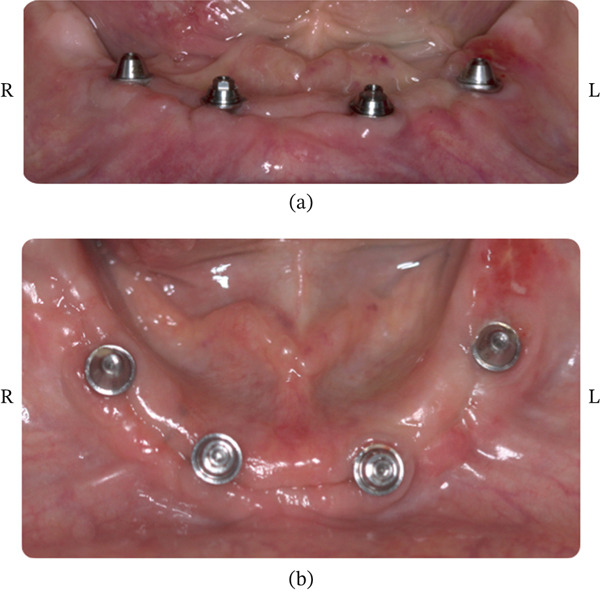
Patient #1 (30 days after operation). (L = left side; R = right side). (a) The frontal image shows a similar healing process between the right and left sides. (b) Occlusal view showing full flap closure without fibrin zones (R) and full flap closure with thin fibrin zones (L).

Clinical observation with EHI was performed at 7, 15, and 30 days after surgery on both the right and left sides (Table 1). The patient received the oral gel only on the right side:

• Day 7 (Figure 9): The right side scored an EHI of 2, with complete flap closure and a fine fibrin line (Figure 9b,c). The left side scored an EHI of 3, with complete flap closure but with larger fibrin clots and some bleeding points (Figure 9b–d).
• Day 15 (Figure 10): The right side showed better healing than the left side (Figure 10a,b). The right side scored an EHI of 1 (complete flap closure, no fibrin zones), while the left side scored an EHI of 2 (complete flap closure with thin fibrin zones) (Figure 10b).
• Day 30 (Figure 11): Healing was similar between the sides; however, the right side, where the gel was applied, showed a smaller healing line (Figure 11a,b). The right side scored an EHI of 1 (complete flap closure, no fibrin areas), while the left side scored an EHI of 2 (complete flap closure with fine fibrin lines and still noticeable wound edges) (Figure 11b).

**Table 1:** EHI scores for Patient #1 at 7, 15, and 30 days after surgery.

Postoperative day	EHI right side (with oral gel)	EHI left side
7 days	2	3
15 days	1	2
30 days	1	2

##### 2.3.1.2. Healing of Patient #2

**Figure 12:**
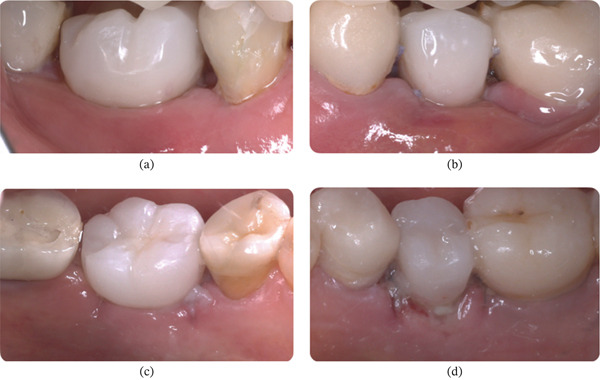
Patient #2 (3 days after operation) ([a, b] right side; [c, d] left side). (a) Lateral buccal view showing complete flap closure with a fine fibrin line in the interproximal area. (b) Lateral lingual view showing complete flap closure with a fine fibrin line in the interproximal area, more evident in the mesial. (c) Lateral buccal view showing complete flap closure with a fine fibrin line in the interproximal area. (d) Lateral lingual view showing incomplete flap closure with fibrin clot in the lingual and interproximal areas.

**Figure 13:**
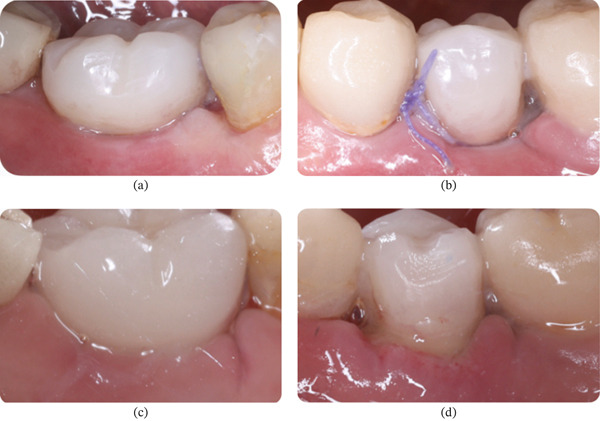
Patient #2 (7 days after operation) ([a, b] right side; [c, d] left side). (a) Lateral buccal view showing complete flap closure with a fine fibrin line in the interproximal area. (b) Lateral lingual view showing complete flap closure with a fine fibrin line in the interproximal area, more evident in the mesial. (c) Lateral buccal view showing complete flap closure with a fine fibrin line in the interproximal area. (d) Lateral lingual view showing incomplete flap closure with fibrin clot in the lingual and interproximal areas.

**Figure 14:**
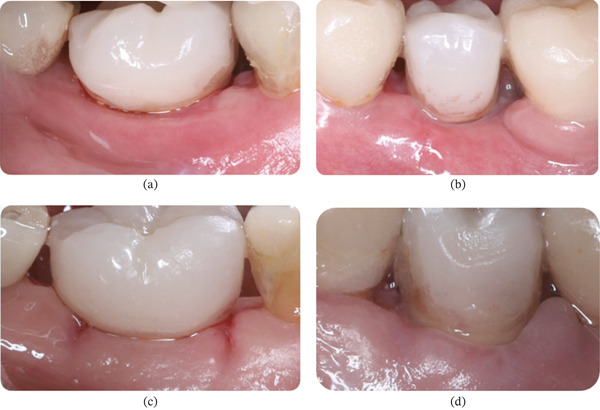
Patient #2 (15 days after operation) ([a, b] right side; [c, d] left side). (a) Better healing was observed on the right side. Lateral buccal view showing complete flap closure and no fibrin line in the interproximal area and marginal region. (b) Lateral lingual view showing complete flap closure and no fibrin line in the interproximal area and marginal region. (c) Lateral buccal view showing complete flap closure with a fine fibrin line in the interproximal area. (d) Lateral lingual view showing incomplete flap closure with a fine fibrin line in the lingual and the interproximal areas.

**Figure 15:**
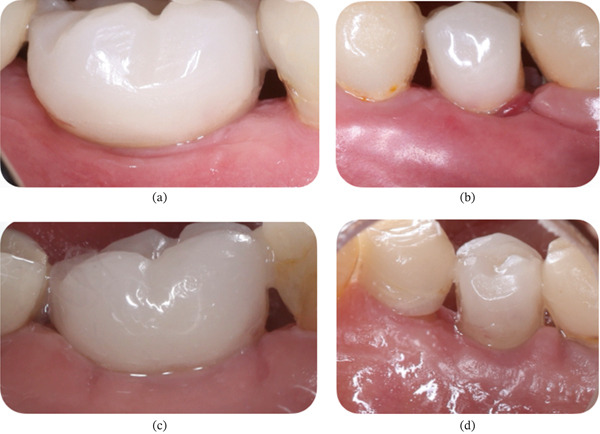
Patient #2 (30 days after operation) ([a, b] right side; [c, d] left side). (a) Lateral buccal view showing total flap closure without fibrin. (b) Lateral lingual view showing total flap closure without fibrin. (c) Lateral buccal view showing complete closure of the flap and fibrin line marginal to the crown, with the presence of inflammation due to bacterial biofilm. (d) Lateral lingual view showing complete closure of the flap and fibrin line marginal to the crown.

Clinical observation with EHI was performed at 3, 7, 15, and 30 days after surgery on both the right (buccal and lingual) and left (buccal and lingual) sides (Table 2). The patient received the oral gel only on the right side:

• Day 3 (Figure 12): On the right side, an EHI of 2 was observed on both the vestibular and lingual sides (Figure 12a,b). On the left side, the vestibular side scored an EHI of 2 (Figure 12c), while the lingual side scored an EHI of 3, with incomplete flap closure and wide fibrin clots (Figure 12d).
• Day 7 (Figure 13): The right side showed visibly better healing. The vestibular side scored an EHI of 1, and the lingual side scored an EHI of 2 (Figure 13a,b). On the left side, the vestibular side scored an EHI of 2, and the lingual side scored an EHI of 3 (Figure 13c,d).
• Day 15 (Figure 14): Better healing was observed on the right side. The right side (vestibular and lingual) scored an EHI of 1 (Figure 14a,b). The left side (vestibular and lingual) scored an EHI of 2 (Figure 14c,d).
• Day 30 (Figure 15): The right side (vestibular and lingual) scored an EHI of 1 (Figure 15a,b). The left side (vestibular and lingual) scored an EHI of 2, with a fibrin line marginal to the crown. Inflammation due to bacterial biofilm was observed on the vestibular side of the left implant.

**Table 2:** Clinical observation with EHI in 3, 7, 15, and 30 days after surgery on the right and left sides in Patient #2.

Postoperative day	EHI right buccal side (with oral gel)	EHI right lingual side (with oral gel)	EHI left buccal side	EHI left lingual side
3 days	2	2	2	3
7 days	1	2	2	3
15 days	1	1	2	2
30 days	1	1	2	2

At all postoperative follow‐ups, the right side showed a better healing process compared to the left side, where the active oxygen gel was not applied.

## 3. Discussion

After implant placement, bacterial control is necessary, as microspaces and infiltration of oral bacteria can lead to colonization of peri‐implant tissues, potentially affecting osseointegration and influencing early implant loss [12]. Wound healing without bacterial intervention is a key point for initial implant success [13]. In parallel, many wound healing processes, such as the oxidative destruction of bacteria, reepithelialization, angiogenesis, and collagen synthesis, are dependent on oxygen. Topical oxygen application to a wound has been shown to promote healing through various processes, such as antibacterial activities, neovascularization, collagen production, epithelialization, phagocytosis, and degradation of necrotic wound tissue [14]. Topical oral oxygen therapy (TOOT) aims to activate the healing process by supporting neovascularization, removing toxins, stimulating the formation of new blood cells, increasing stem cell production, and killing bacteria [15]. This effect can be observed in the clinical cases presented, where the side that received topical oxygen therapy showed better healing than the side that did not. In this way, we can suggest that the BlueM oral gel led to faster healing, as has been shown in previously published studies [8].

In this descriptive and observational case report, the healing of postsurgical wounds with and without the use of active oxygen gel (BlueM) was compared by clinical observation and described using the EHI from a previous research article [11, 13]. This index not only differentiates between degrees of tissue exposure but also records the amount of fibrin formation when complete closure is present. According to the authors [11], clinical experience has shown that the most rapid and uneventful healing is associated with no or minimal fibrin formation, as this occurs when surgical trauma has been kept to a minimum. This phenomenon was observed in these clinical cases. In Patient #1, where full‐arch surgery was performed, a smaller scar line was present where the active oxygen‐releasing gel was used (right side). This suggests that the gel helped stimulate faster healing with less fibrin formation.

Another important point is that TOOT with BlueM may be a safe alternative to chlorhexidine (CHX) in postsurgical care, actively promoting healing processes while also acting as an antiseptic to effectively reduce microbial load and pain during the postoperative period [16]. It is suggested that the use of the oral gel may have contributed to less local biofilm accumulation due to its bactericidal action. This can be observed 30 days after surgery in Patient #2, where the left side that did not receive the oral gel showed a greater accumulation of biofilm and the presence of inflammation.

CHX is considered the gold standard in the antiseptic treatment of the oral mucosa because of its broad antibacterial spectrum [17]. However, studies have shown that CHX has a cytotoxic effect on gingival fibroblasts [18], gingival epithelial cells [19], periodontal ligament cells [20], and cultured alveolar and osteoblastic cells, interfering with tissue regeneration processes [21, 22]. Although CHX has proven bactericidal activity, these factors can interfere with the healing process. Thus, new product options like BlueM oral care, which offer both biofilm control and stimulation of the healing process via topical oxygen release, are available for clinical and surgical procedures, presenting good clinical [10, 23] and scientific results [8, 9, 24]. This can be observed in the healing process of the clinical cases presented here.

## 4. Conclusion

Based on the comparative observation in both clinical cases, the use of active oxygen in an oral gel (BlueM) is suggested to act as both a bactericidal action and a healing stimulant for oral surgical wounds. These cases indicate it is a reliable alternative for the postsurgical care of oral wounds after the placement of dental implants, where it appeared to optimize wound healing. Further controlled clinical studies are required to substantiate these findings and address the limitations of these cases.

## Author Contributions

**Reyna María Ocegueda Estrada**: investigation, data collection, clinical support, writing – review and editing. **Fabio Luiz Andretti**: formal analysis, writing – review and editing. **Tatiana Miranda Deliberador**: conceptualization, methodology, data curation, writing – original draft, writing – review and editing, supervision.

## Funding

No funding was received for this manuscript.

## Disclosure

All authors have read and approved the final version of the manuscript. The corresponding author had full access to all of the data in these cases and takes complete responsibility for the integrity of the data and the accuracy of the data analysis.

## Consent

This manuscript presents a clinical case report. Written informed consent was obtained from all patients, authorizing the use of their clinical data and images for publication purposes, in accordance with ethical standards. The patients signed written consent forms regarding the publishing of their data and photographs.

## Conflicts of Interest

Professor Tatiana Deliberador is an academic and scientific researcher from BlueM Brazil and receives monthly fees from the company. However, this conflict of interest did not influence the results found in the article. BlueM did not fund the execution of these clinical cases. However, it will pay the publication fee.

## Data Availability

The authors confirm that the data supporting the findings of these cases are available within the article.
